# Trigeminal Nerve Transection-Induced Neuroplastic Changes in the Somatosensory and Insular Cortices in a Rat Ectopic Pain Model

**DOI:** 10.1523/ENEURO.0462-18.2019

**Published:** 2019-01-28

**Authors:** Satoshi Fujita, Kiyofumi Yamamoto, Masayuki Kobayashi

**Affiliations:** 1 Department of Pharmacology, Nihon University School of Dentistry, Chiyoda-ku, Tokyo 101-8310, Japan; 2 Division of Oral and Craniomaxillofacial Research, Dental Research Center, Nihon University School of Dentistry, Chiyoda-ku, Tokyo 101-8310, Japan; 3 Molecular Imaging Research Center, RIKEN, Chuo-ku, Kobe 650-0047, Japan

**Keywords:** somatosensory cortex, insular cortex, nociception, dental pulp, somatotopy

## Abstract

The primary sensory cortex processes competitive sensory inputs. Ablation of these competitive inputs induces neuroplastic changes in local cortical circuits. However, information concerning cortical plasticity induced by a disturbance of competitive nociceptive inputs is limited. Nociceptive information from the maxillary and mandibular molar pulps converges at the border between the ventral secondary somatosensory cortex (S2) and insular oral region (IOR); therefore, S2/IOR is a suitable target for examining the cortical changes induced by a disturbance of noxious inputs, which often causes neuropathic pain and allodynia. We focused on the plastic changes in S2/IOR excitation in a model of rats subjected to inferior alveolar nerve transection (IANX). Our optical imaging using a voltage-sensitive dye (VSD) revealed that the maxillary molar pulp stimulation-induced excitatory propagation was expanded one to two weeks after IANX at the macroscopic level. At the cellular level, based on Ca^2+^ imaging using two-photon microscopy, the amplitude of the Ca^2+^ responses and the number of responding neurons in S2/IOR increased in both excitatory and inhibitory neurons. The *in vitro* laser scanning photostimulation (LSPS) revealed that Layer II/III pyramidal and GABAergic fast-spiking neurons in S2/IOR received larger excitatory inputs from Layer IV in the IANX models, which supports the findings obtained by the macroscopic and microscopic optical imaging. Furthermore, the inhibitory postsynaptic inputs to the pyramidal neurons were decreased in the IANX models, suggesting suppression of inhibitory synaptic transmission onto excitatory neurons. These results suggest that IANX induces plastic changes in S2/IOR by changing the local excitatory and inhibitory circuits.

## Significance Statement

The inferior alveolar nerve (IAN), a mandibular branch of the trigeminal nerve, innervates the orofacial region, including the tooth pulps. IAN transection (IANX) induces allodynia in the maxillary nerve-projecting region. This study demonstrates the facilitation of excitatory propagation in the secondary somatosensory cortex (S2) and insular oral region (IOR) following maxillary molar tooth pulp stimulation in IANX rats. Both pyramidal and fast-spiking neurons in Layer II/III S2/IOR of IANX rats received larger glutamatergic excitatory inputs from the deeper layers. Reduction of GABA_A_ receptor-mediated IPSCs in pyramidal neurons of IANX rats may contribute to the facilitated excitation in S2/IOR. These findings suggest that GABAergic neurons in S2/IOR could be a therapeutic target for ectopic pain in the orofacial region.

## Introduction

The dental pulp is an appropriate organ for studying the neural mechanisms of nociception because most neural fibers in the dental pulp are classified as Aδ and C, which transmit nociception information to the central nervous system, and few Aβ fibers are involved (Naftel et al., 1999). Nociceptive information in the dental pulp is principally conveyed to the trigeminal spinal subnucleus of caudalis (Sp5C; medullary dorsal horn; Marfurt and Turner, 1984). Subsequently, the parabrachial nucleus and thalamic nuclei, including the ventral posteromedial nucleus (VPM) and intralaminar nucleus, receive nociceptive inputs (Sato et al., 2017). Nociceptive information is further transmitted to the ventral part of the primary somatosensory cortex (S1) and secondary somatosensory cortex (S2) and the dorsal part of the insular oral region (IOR) in the rat (Shigenaga et al., 1974; Remple et al., 2003; Nakamura et al., 2015, 2016). Our previous optical imaging and extracellular recording studies revealed that S2/IOR exhibits somatotopic organization in response to electrical pulpal stimulation of the mandibular and maxillary incisors and molars (Nakamura et al., 2015, 2016). In addition, the systemic administration of potent analgesics, i.e., morphine, effectively suppresses excitation in S2/IOR in response to pulpal stimulation (Kaneko et al., 2017), suggesting that S2/IOR processes nociceptive information in the dental pulp.

The inferior alveolar nerve (IAN) innervates the mandibular teeth, gingiva, lower lip, and mentum and carries somatosensory information, including information regarding touch and pressure senses, nociception, and thermal sensation. IAN injury could be caused by clinical dental treatments, such as wisdom tooth extraction and sagittal split ramus osteotomy, causing not only severe sensory paralysis in the mandibular region but also neuropathic pain, such as allodynia and hyperalgesia (Renton and Yilmaz, 2011; Renton et al., 2012). Referred pain in the orofacial regions is another complication; patients with an injured IAN perceive pain originating from the site of dental (pulpal) pain in other regions innervated by the maxillary nerve (Bender, 2000). Some of the mechanisms in the primary and secondary neurons in the trigeminal nervous system have been elucidated. Iwata and his colleagues have demonstrated that the intensive neural activities of primary neurons are transmitted to adjacent primary neurons in the trigeminal ganglion (TG) by releasing a growth factor (Shinoda et al., 2011; Matsuura et al., 2013; Ohara et al., 2013). In addition to primary neurons, secondary neurons are involved in ectopic mechanical allodynia as follows: fractalkine signaling activates microglia to release IL-1β onto secondary trigeminal neurons, which increases their excitability (Kiyomoto et al., 2013). These findings suggest that a disturbance of somatosensory input from the mandibular branch of the trigeminal nerve induces long-term changes in somatosensation in orofacial regions innervated by other branches, such as the maxillary nerve. However, it remains unclear if the cerebral cortex, where sensory information is finally processed, is involved in referred dental pain.

Multiple sensory information inputs converge in the primary sensory cortex. For example, the primary visual cortex receives optical information from both the ipsilateral and contralateral eyes, forming competitive synaptic inputs (Hubel and Wiesel, 1970). The influence of the ablation of components of competitive inputs on the sensory cortex has been extensively explored, revealing that neuroplastic changes clearly occur in local circuits in the visual, auditory, and somatosensory cortices (Feldman, 2009). Although nociceptive information from the maxillary and mandibular molar pulps converges at the border between the ventral S2 and IOR, there is limited information concerning cortical plasticity induced by a disturbance in competitive nociceptive inputs. The plastic changes in S2/IOR in response to IAN transection (IANX) may reflect the underlying mechanism of neuropathic pain, including referred pain, because ectopic pain often continues even after the recovery of primary afferents. Therefore, in the present study, we examined the cerebral cortex in an IANX animal model and pursued the neural mechanism of plastic changes induced by IANX.

## Materials and Methods

The experiments performed in this study were approved by the Animal Experimentation Committee of Nihon University and were conducted in accordance with institutional guidelines for the care and use of experimental animals as described in the National Institute of Health Guide for the Care and Use of Laboratory Animals. All efforts were made to minimize animal suffering and reduce the number of animals used.

### IANX model

Fifty-eight male Sprague Dawley rats (Japan SLC) were used in the voltage-sensitive dye (VSD) imaging experiments. Vesicular GABA transporter (VGAT)-Venus line A and B transgenic rats (*N* = 58 and *N* = 3, respectively) in which ∼98% and 95% of cortical GABAergic cells are fluorescently labeled (Nagai et al., 2002; Uematsu et al., 2008) were used for the two-photon *in vivo* imaging and *in vitro* whole-cell patch-clamp experiments, respectively. All rats were housed in clear polycarbonate cages (length × width × height = 48 × 26.5 × 21 cm) containing paper shavings as bedding and kept in a temperature-controlled room (23 ± 2°C) on a 12/12 h light/dark cycle with *ad libitum* access to food and water.

**Figure 1.**
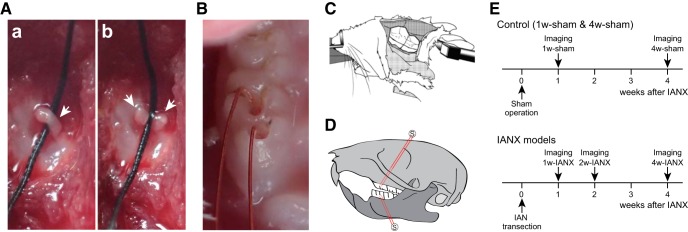
Experimental design of the VSD imaging experiment. ***A***, IAN (arrows) in the right mandibular bone was exposed (***a***) and transected (***b***) in a model that received IANX. In the photographs, a black silk thread was placed under the IAN to clearly show the IAN. ***B***, To stimulate the molar pulps, enamel-coated copper wire electrodes were inserted into the maxillary and mandibular 1st molar pulps. ***C***, A schematic drawing of the *in vivo* preparation for the optical imaging of the somatosensory and insular cortices. ***D***, Position of the stimulation electrodes (maxillary and mandibular 1st molars). ***E***, Schedule of operation and VSD imaging. The sham operations or IANXs were performed at postnatal weeks 5–6. In the sham groups, VSD imaging was performed one week (1w-sham) or four weeks (4w-sham) after the sham operation. In the IANX models, the VSD imaging was performed one week (1w-IANX), two weeks (2w-IANX), or four weeks (4w-IANX) after IANX.

IANX was performed at five to six weeks of age under general anesthesia with isoflurane (2%; Mylan) following a subcutaneous injection of the local anesthetic lidocaine (2%; Mylan; Fig. 1*A*). A part of the right mandibular bone immediately above the angle of the mandible was removed to expose the IAN, which was transected, including the mental nerve. After IANX, the IAN was repositioned in the mandibular canal without a gap between the transected nerve ends. The incisions were closed using 6-0 silk sutures.

As a control, we used sham-operated rats that received a sham operation identical to that described above without IANX one week (1w-sham) or four weeks (4w-sham; Fig. 1*E*) after IANX. Optical imaging using a VSD was performed using the rats one (1w-IANX), two (2w-IANX), or four (4w-IANX) weeks after IANX. The Ca^2+^ imaging and whole-cell patch-clamp recordings were performed using the 1w-sham and 1w-IANX rats.

### *In vivo* optical imaging using a VSD

**Figure 2.**
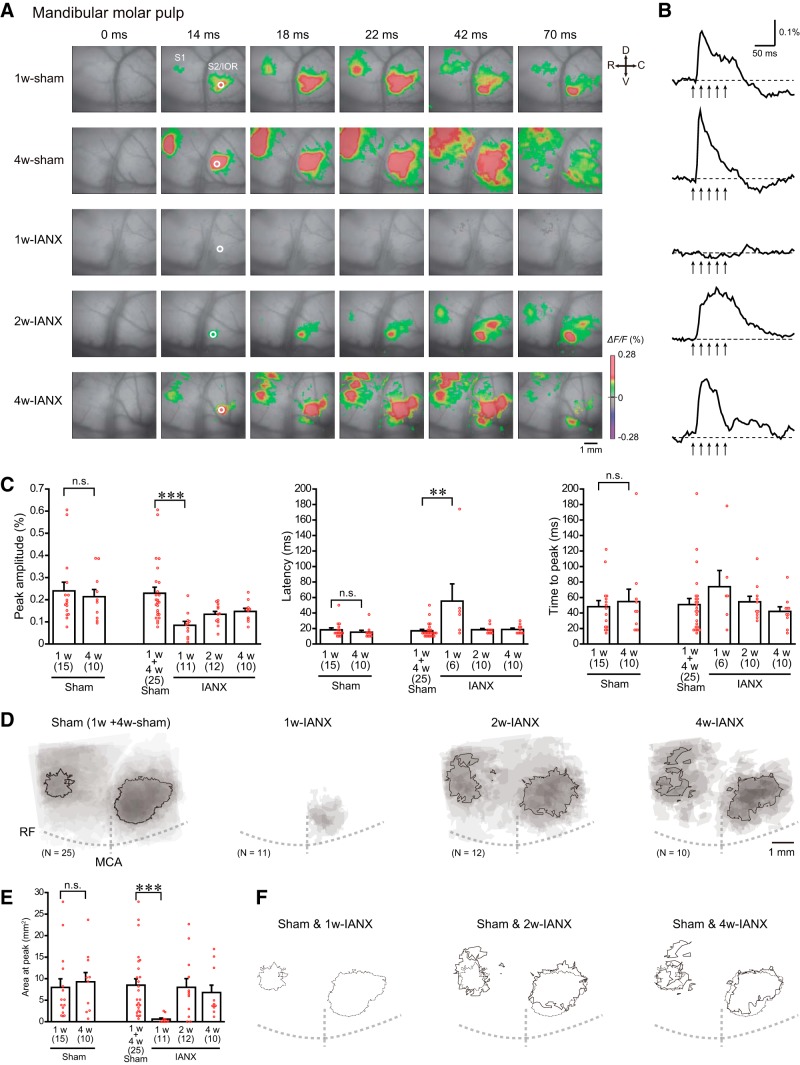
IANX transiently decreased the cortical responses to the mandibular molar pulp stimulation. ***A***, Examples of cortical responses evoked by the electrical stimulation of the mandibular molar pulp in the Sham (1w-sham and 4w-sham) and IANX (1w-IANX, 2w-IANX, and 4w-IANX) groups. The time from the onset of the electrical stimulation is shown on the top of each panel. R, rostral; C, caudal; D, dorsal; V, ventral. ***B***, Temporal profiles of optical signals in the ROIs (white circles) in S2/IOR shown in ***A***. Arrows indicate the timing of the electrical stimulation. ***C***, Comparison of the peak amplitude, latency, and time to peak of optical signals in response to electrical stimulation of the mandibular molar pulp. The numbers of animals are shown in parenthesis. Note that the latency and time to peak were estimated in cases in which significant optical signals were obtained. There was no significant difference between 1w-sham and 4w-sham groups (n.s.; Mann–Whitney *U* test). ***D***, Superimposed images of the maximum responses evoked by the mandibular molar pulp stimulation. The number of overlapping responses is represented by gradation of the color density. The line outlines the area responding to stimulation in 50% of animals. RF, rhinal fissure. ***E***, Comparison of areas activated by the stimulation. Note the significant decrease in the excitation area in the 1w-IANX group (Mann–Whitney *U* test). ***F***, Comparison of the excitation area shown in ***D***. Solid and dashed lines indicate the responding area in 50% of the animals of the IANX model and sham groups, respectively. Note that the only outlines of the sham group are shown in the left panel because of no superimposed area in 50% rats of 1w-IANX group; **p < 0.01, ***p < 0.001, Mann–Whitney *U* test.

**Figure 3.**
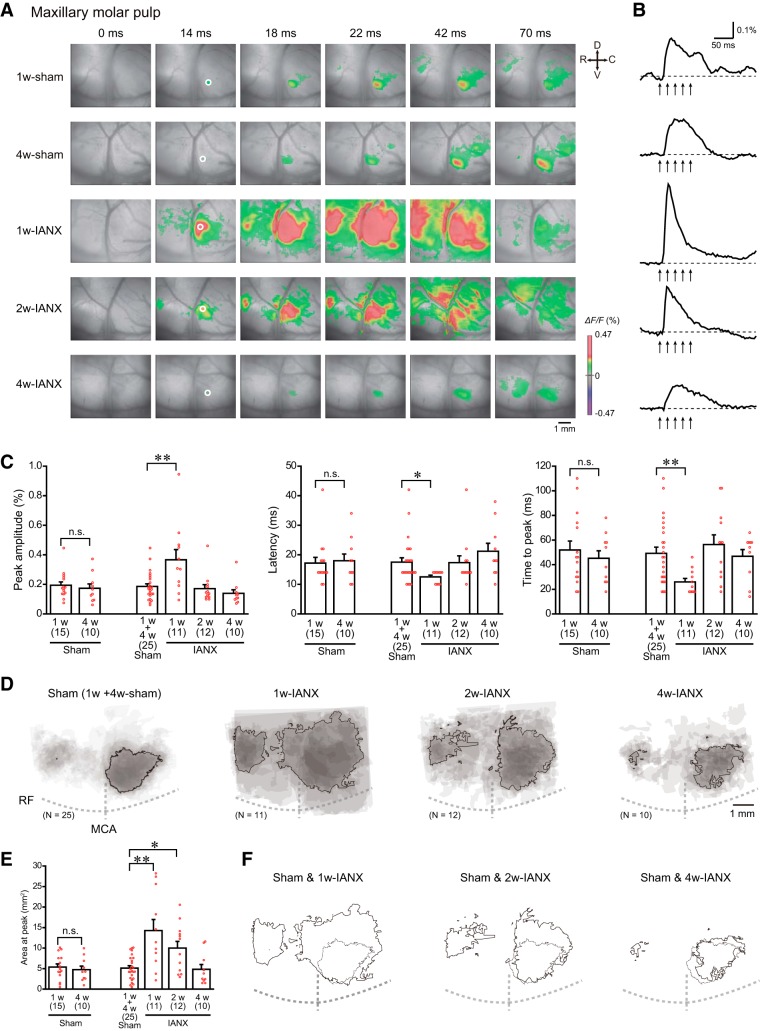
IANX transiently increased cortical responses to maxillary molar pulp stimulation. ***A***, Examples of cortical responses evoked by electrical stimulation of the maxillary molar pulp in the sham (1w-sham and 4w-sham) and IANX model (1w-IANX, 2w-IANX, and 4w-IANX) groups. The time from the onset of the electrical stimulation is shown at the top of each panel. ***B***, Temporal profiles of optical signals in ROIs (white circles) in S2/IOR shown in ***A***. ***C***, Comparison of the peak amplitude, latency, and time to peak of optical signals responding to the stimulation. The numbers of animals are shown in parenthesis. Note that there was no significant difference between the 1w-sham and 4w-sham groups (Student’s *t* test or Mann–Whitney *U* test). ***D***, Superimposed images of the maximum responses evoked by the maxillary molar pulp stimulation. The number of overlapping responses is represented by the gradation of the colors. The line outlines the area responding to stimulation in 50% of animals. ***E***, Comparison of areas activated by the stimulation. Note the significant increases in the excitation area in the 1w-IANX and 2w-IANX groups (Mann–Whitney *U* test). ***F***, Comparison of the excitation area shown in ***D***. Solid and dashed lines indicate the responding area in 50% of the animals in the IANX model and sham groups, respectively. Note that the activated area expanded primarily dorsally in the 1w-IANX and 2w-IANX groups; *p ≤ 0.017, **p < 0.01, Mann–Whitney *U* test.

We performed optical imaging using a VSD (RH1691, Optical Imaging) as previously described (Horinuki et al., 2015, 2016; Nakamura et al., 2015, 2016; Fujita et al., 2016, 2017; Yokota et al., 2016; Kobayashi and Horinuki, 2017) to observe IANX-induced changes in the spatiotemporal profiles of the excitatory propagation induced by electrical stimulation of the dental pulps at the macroscopic level (Figs. 1-3). Briefly, male Sprague Dawley rats were anesthetized with urethane (1.5 g/kg, i.p.), in combination with atropine methyl bromide (5 mg/kg, i.p.). The toe pinch reflex was monitored to maintain an adequate depth of anesthesia, and urethane was added as needed. The body temperature was maintained at ∼37°C using a rectal probe and a heating pad (BWT-100, Bio Research Center). Lidocaine (2% gel, AstraZeneca) was applied to the incisions to ensure complete analgesia. Each animal was fixed to a stereotaxic frame to image the surface of the left IC using a CCD camera (MiCAM02, Brainvision). A craniotomy was performed to expose the left IC and surrounding cortices (Fig. 1*C*).

RH1691 (1 mg/ml) was loaded onto the cortical surface for 1 h. The fluorescent changes in RH1691 were obtained by a CCD camera system mounted on a stereomicroscope (Leica Microsystems). The cortical surface was illuminated through a 632-nm excitation filter and a dichroic mirror by a tungsten-halogen lamp (CLS150XD, Leica Microsystems), and the fluorescent emission was captured through an absorption filter (λ > 650 nm longpass, Andover). The CCD camera had a 6.4 × 4.8 mm^2^ imaging area (184 × 124 pixels).

Bipolar enamel-coated copper wire electrodes (diameter = 80 μm) were inserted into the maxillary and mandibular 1st molar pulps (Fig. 1*B*,*D*). Electrical stimulation (100-μs duration, 5 V) was applied using a stimulator unit (STG2008, MultiChannel Systems). To obtain stable optical responses, five voltage pulses (50 Hz) were applied at 0.05 Hz.

To compensate for the acute bleaching of the dye, each image showing a response to the dental pulp stimulation was subtracted from the image without stimulation. The sampling rate was 250 Hz, and 32 consecutive images of responses to stimuli were averaged.

The changes in the intensity of the fluorescence (*ΔF*) of each pixel were divided by the initial fluorescence intensity (*F*), and the ratio (*ΔF*/*F*) was processed with a spatial filter (9 × 9 pixels). A significant response was defined as a signal exceeding seven times the SD of the baseline noise. The optical imaging data were processed and analyzed using the Brain Vision Analyzer (Brainvision). The images were aligned across multiple rats using the rhinal fissure and middle cerebral artery (MCA) as markers. Rats with angioplany of the MCA, e.g., bifurcation at the rhinal fissure, were excluded from the analyses because the rhinal fissure and the MCA could not be aligned with those of the other animals.

We estimated the spatiotemporal profiles of excitation based on the initial and maximum responses. We used the initial response that first exhibited a significant increase in the optical signal to define the region of interest (ROI) for the analysis of the temporal profiles of the optical signal. In some cases, the initial responses were found simultaneously in both S1 and S2/IOR. Since our previous study indicated that the S2/IOR plays a critical role in the processing of nociceptive information (Kaneko et al., 2017), all ROIs were set based on the initial responses in S2/IOR. The peak amplitude was defined as the maximum amplitude of an optical response at an ROI. The latency and time to peak were defined as the time from the onset of stimulation to the detection of a significant optical response and to the time of the peak amplitude, respectively. In the estimation of the latency and time to peak, we excluded the cases that did not show a significant response. To estimate the spatial profiles, the maximum response was defined as the outline of the excitatory response in the frame with the peak amplitude of optical signal in the center of the initial response.

### Ca^2+^ imaging *in vivo*

**Figure 4.**
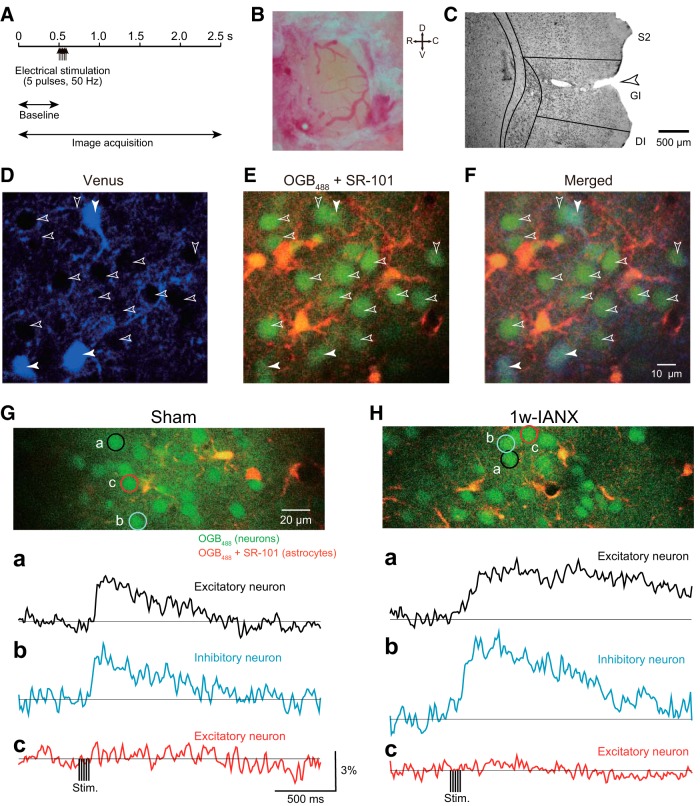
Ca^2+^ imaging by two-photon microscopy. ***A***, Stimulation and image acquisition protocol used for the Ca^2+^ imaging. ***B***, An example of craniotomy in S2/IOR. The diameter was 0.5–1.0 mm. ***C***, An example of a recorded region. After the Ca^2+^ imaging, the recorded region was marked by penetration with a heated needle. Note that the location of the recorded region was set around the border between S2 and IC. ***D***, An example of imaged fluorescence of Venus. Before the injection of a mixture of OGB_488_, which is a Ca^2+^ indicator, and SR-101, which is an astrocyte marker, the distribution of Venus-positive neurons in VGAT-Venus rats was systematically imaged (excitation wavelength = 950 nm). Green fluorescence was artificially recolored to blue in this figure. ***E***, An example of imaged florescence of OGB_488_ and SR-101 (excitation wavelength = 800 nm). Note that the neurons exhibited green fluorescence only, and glial cells exhibited both green and red fluorescence. ***F***, *Post hoc* identification using merged image of Venus (***D***) and OGB_488_/SR-101 (***E***). Note that the open and filled arrowheads indicate Venus-negative (non-GABAergic; excitatory) and Venus-positive (GABAergic; inhibitory) neurons, respectively. ***G***, An example of Ca^2+^ responses to electrical stimulation of the maxillary molar pulp in a sham rat. The imaged area is shown in the top panel. The circles (***a***, ***b***, ***c***) indicate the ROIs. Traces of Ca^2+^ signals in the responding excitatory (***a***) and inhibitory (***b***) neurons and a nonresponding excitatory neuron (***c***) are shown in the lower panels. ***H***, An example of Ca^2+^ responses to electrical stimulation of the maxillary molar pulp in an IANX rat. IANX was performed one week before the Ca^2+^ imaging. The imaged area is shown in the top panel. Temporal changes in Ca^2+^ signals in responding excitatory (***a***) and inhibitory (***b***) neurons and a nonresponding excitatory neuron (***c***) are shown in the lower panels. Note that the Ca^2+^ responses in the responding neurons in the IANX rat were long lasting compared to those in the sham rat. DI, dysgranular IC; GI, granular IC.

VGAT-Venus line A rats and B rats (*N* = 22 and *N* = 3, respectively) were used for the two-photon Ca^2+^ imaging experiment. Under urethane anesthesia (as described above), a small craniotomy (diameter of 0.5–1.0 mm; Fig. 4*B*) was performed on a part of the S2/IOR region (Fig. 4*C*). The dura matter was carefully resected. The MCA and rhinal fissure were visualized by thinning the skull and were used as landmarks to determine the location of the craniotomy. Two-photon imaging was performed using a laser-scanning microscope system (FVMPE-RS, Olympus) equipped with an upright microscope (BX63, Olympus), a 25× water immersion objective (N.A. = 1.05; XLPLN25XWMP2, Olympus), and a pulsed laser (Insight DS Dual-OL, Spectra Physics). Before the injection of the Ca^2+^ indicator, the fluorescence of Venus was systematically imaged with a 254 × 254 μm field of view consisting of 512 × 512 pixels from the cortical surface to 400 μm at 1-μm intervals (excitation wavelength = 950 nm; Fig. 4*D*) for the *post hoc* identification of Venus-negative (excitatory glutamatergic) and Venus-positive (inhibitory GABAergic) neurons. A Ca^2+^ indicator, i.e., Oregon Green 488 BAPTA-1 AM (OGB_488_; Invitrogen), was dissolved in 20% pluronic F-127 in DMSO (Invitrogen) and diluted in Ringer’s solution containing the astrocyte marker sulforhodamine 101 acid chloride (SR-101; Dojindo). A micropipette (inner diameter at the tip = ∼7 μm) made from a glass capillary (GC150TF-10, Harvard Apparatus) using a Flaming/Brown micropipette puller (P-97, Sutter Instruments) was filled with a solution containing 0.8 mM OGB_488_, 0.1 mM SR-101, 1% pluronic F-127, and 5% DMSO. This solution was injected into the targeted area (depth = 200–300 μm) by applying air pressure (0.2–2.0 psi) for 10 min. The exposed cortex was covered with 2% agarose (Agarose L; Nippon Gene) dissolved in Ringer’s solution with a slip of cover glass 30 min after the OGB_488_/SR-101 injection. To confirm the loading of the Ca^2+^ indicator and astrocyte marker, we imaged the same region of Venus fluorescence that was imaged before the dye injection (Fig. 4*E*).

The Ca^2+^ responses to the electrical stimulation of the maxillary molar pulp were recorded. The stimulation method was the same as that described above for the VSD imaging (Fig. 4*A*), except for the intensity of the electrical stimulation (7 V was applied in the Ca^2+^ imaging). The density of the neurons was used to distinguish Layer II/III from Layer I (Takata and Hirase, 2008). The fluorescence of OGB_488_ and SR-101 in Layer II/III was excited with an 800-nm wavelength laser, and the emitted light was split into red and green light by a dichroic mirror (570 nm) with bandpass filters (495–540 nm for green, 575–645 nm for red; FV30-FGR, Olympus). The imaged area was a 254 × 72 μm field of view consisting of 512 × 144 pixels. The sampling rate was 100 Hz, and 20 consecutive images of the response to the stimuli were averaged.

After the recordings, the rats were deeply anesthetized with 5.0% isoflurane and perfused through the ascending aorta with saline, followed by 300 ml of a ﬁxative containing 4% paraformaldehyde in 0.1 M PB (pH 7.4). The brains were removed, postﬁxed overnight, and cryoprotected in 30% sucrose in 0.1 M PB. The brains were frozen, sectioned coronally at 50 μm, and stained with 0.25% thionine to examine the recording site (Fig. 4*C*).

**Figure 5.**
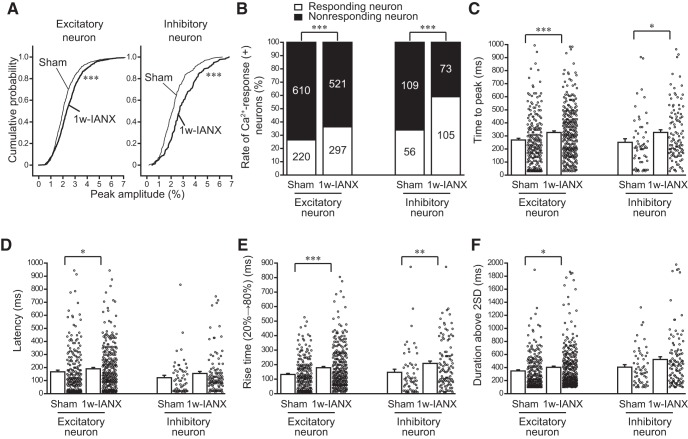
Ca^2+^ responses to maxillary molar pulp stimulation obtained from sham (*N* = 14) and 1w-IANX (*N* = 11) rats. ***A***, Cumulative curves of the peak amplitude in excitatory and inhibitory neurons. Note that the number of neurons showing a high peak amplitude increased in both types of neurons in the IANX rats; ***p < 0.001, Kolmogorov–Smirnov test. ***B***, Rates of responding neurons. The number of neurons is shown in the columns; ***p < 0.001, χ^2^ test. ***C–F***, Time to peak (***C***), latency (***D***), 20–80% rise time (***E***), and duration above 2 SD (***F***) are shown; *p < 0.05, **p < 0.01, ***p < 0.001, Mann–Whitney *U* test.

We distinguished the neurons from glial cells by the fluorescence of SR-101. The neurons exhibited only green fluorescence while the astrocytes exhibited both green and red fluorescence (Fig. 4*E*). The distinguished neurons were further divided into excitatory and inhibitory neurons by comparing the images before and after the injection of OGB_488_/SR-101 (Fig. 4*F*). To minimize contamination of Ca^2+^ signals from neuropil, we excluded neurons whose somata overlapped neural fibers in the same slice image by scanning the Venus fluorescence. The ratio *ΔF/F* was obtained by dividing the changes in the intensity of the fluorescence (*ΔF*) of each pixel by the initial fluorescence intensity (*F*) and processed with a spatial filter (9 × 9 pixels) and a cubic filter (3 × 3 pixels and three time frames). The Ca^2+^ imaging data were processed and analyzed using the software program Brain Vision Analyzer (Brainvision; Fig. 5). The time to peak was defined as the time between the onset of stimulation and the time of the maximum response. The latency was obtained by subtracting the onset of stimulation from the time at which a Ca^2+^ response exceeded twice the SD of the baseline noise before the maximum response. The rise time was obtained from the time in which 20–80% of the peak amplitude was observed. To exclude noise from the evoked responses, significant responses were defined as events whose *ΔF/F* during the period surrounding the time to peak continuously exceeded twice the SD of the baseline noise for at least 100 ms.

### Whole-cell patch-clamp recordings

The VGAT-Venus line A transgenic rats (*N* = 31) were used for the *in vitro* whole-cell patch-clamp recording. The techniques used to prepare the *in vitro* cortical slices were similar to those previously described (Takei et al., 2017; Yamamoto and Kobayashi, 2018). Briefly, the rats were anesthetized with isoflurane (5%) and decapitated. Cortical tissue blocks were cut at a 350-μm thickness using a microslicer (Linearslicer Pro 7, Dosaka EM) in ice-cold modified artificial CSF (ACSF) containing the following: 230 mM sucrose, 2.5 mM KCl, 10 mM MgSO_4_, 1.25 mM NaH_2_PO_4_, 26 mM NaHCO_3_, 0.5 mM CaCl_2_, and 10 mM D-glucose. After a 15-min recovery, the coronal slices were transferred into normal ACSF (pH 7.35–7.40) that contained 126 mM NaCl, 3 mM KCl, 2 mM MgSO_4_, 1.25 mM NaH_2_PO_4_, 26 mM NaHCO_3_, 2 mM CaCl_2_, and 10 mM D-glucose. The modified and normal ACSF were aerated with a mixture of 95% O_2_/5% CO_2_.

The perfusion rate of the normal ACSF in the recording chamber was set at 2.0 ml/min. Whole-cell patch-clamp recording was performed from Layer II/III pyramidal or fast-spiking GABAergic neurons under a voltage-clamp condition (V_H_ = –60 mV). We identified the neuronal subtypes by the spike firing properties and Venus fluorescence by using a confocal microscope equipped with Nomarski optics (BX-61, Olympus) and an infrared-sensitive video camera (C3077-78, Hamamatsu Photonics), as previously reported (Kobayashi et al., 2012; Koyanagi et al., 2014; Yamamoto et al., 2015; Yamamoto and Kobayashi, 2018). The distance between the recorded cells was <100 μm. The electrical signals were recorded with an amplifier (Multiclamp 700B, Molecular Devices) and a digitizer (Digidata 1440A, Molecular Devices), observed on-line, and stored on a computer hard drive using Clampex (pClamp 10, Molecular Devices).

**Figure 6.**
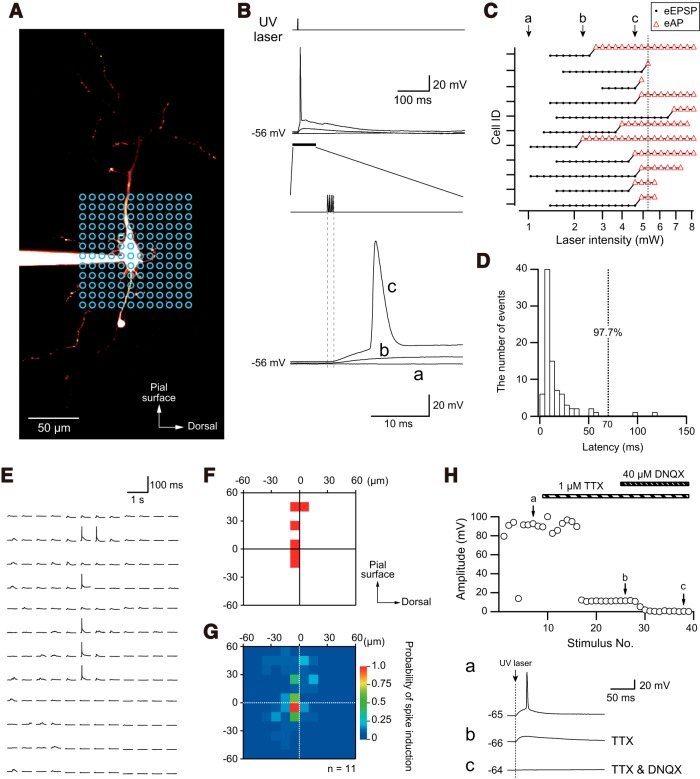
LSPS in the presence of 200 µM caged-glutamate. ***A***, A representative confocal image of a Layer V pyramidal neuron visualized with Alexa Flour 594. UV laser photostimulation sites are indicated by blue circles (10-µm interval). ***B***, Voltage responses evoked by direct photostimulation (5-ns duration, four pulses at 5 kHz) onto the soma shown in ***A***following the application of HDC ACSF containing 200 µM caged-glutamate. Time-expanded traces are shown in the bottom panel. The intensity of LSPS was 1.0 mW (***a***), 2.3 mW (***b***), and 4.6 mW (***c***). Note that depolarization is intensity dependent, and 4.6-mW LSPS evoked a single action potential. ***C***, LSPS intensity-dependent action potential induction in 11 pyramidal neurons from nine rats. Application of LSPS at 5.1 mW to the soma of pyramidal neurons invariably evoked action potentials (eAP). Induction of EPSPs without action potentials is shown by dots (eEPSP). The top neuron is shown in ***A***, ***B***. Traces of ***a***, ***b***, ***c*** in ***B*** are indicated by arrows in ***C***. ***D***, A histogram of the latency of LSPS (5.1 mA)-induced action potentials in pyramidal neurons (*n* = 13). Within 70 ms of the onset of LSPS, 97.7% of pyramidal neurons evoked action potentials. ***E***, ***F***, Voltage responses to LSPS to each circle (***E***) and hot map of LSPS-induced action potentials (***F***, red pixels) in the same neuron shown in ***A***, ***B***. ***G***, A color-coded map showing the probability of action potential induction in 11 pyramidal neurons. Note that the high probability at the soma contradicts the low probability at the distal dendrites. The origin of the coordinates indicates the center of the soma. ***H***, Effects of 1 μM TTX and 40 μM DNQX on evoked responses by LSPS. Action potentials were blocked by TTX, and the residual depolarizing potential was completely diminished by DNQX.

The pipette solution contained 135 mM potassium gluconate, 10 mM HEPES, 0.5 mM EGTA, 2 mM MgCl_2_, 2 mM magnesium ATP, and 0.3 mM sodium GTP. In the IPSC recording experiment, the pipette solution contained 120 mM cesium gluconate, 10 mM HEPES, 8 mM NaCl, 5 mM QX-314, 2 mM magnesium ATP, 0.3 mM sodium GTP, and 0.1 mM BAPTA. The pipette solutions had a pH 7.3 and an osmolarity of 300 mOsm. The liquid junction potentials of the potassium gluconate-based and cesium gluconate-based pipette solutions were –6 and –12 mV, respectively. The voltage was not corrected in the present study. Thin-wall borosilicate patch electrodes (2–5 MΩ) were pulled on a Flaming/Brown micropipette puller (P-97, Sutter Instruments). Alexa Fluor 594 (Invitrogen) was added to the internal solution in a subset of the experiments (Fig. 6).

The recordings were performed at room temperature. The seal resistance was >10 GΩ, and only data obtained from electrodes with access resistance of 6–20 MΩ that showed <20% change during the recordings were included in this study.

### Caged glutamate experiment *in vitro*

**Figure 7.**
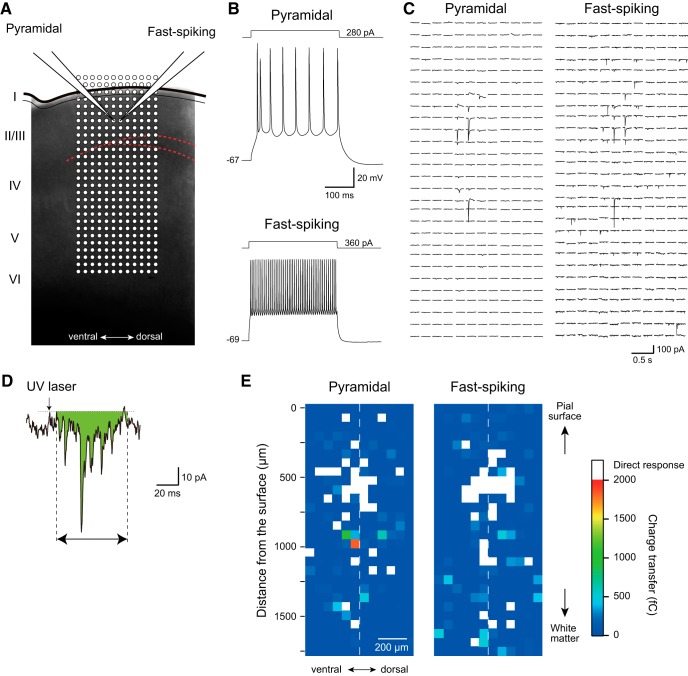
Representative examples of excitation maps of Layer II/III pyramidal and fast-spiking neurons obtained by LSPS. ***A***, Translucent image of an IC slice with two patch electrodes attached to pyramidal and fast-spiking neurons. LSPS sites (open circles; 62.5-µm interval) are superimposed. ***B***, Repetitive firing properties of recorded pyramidal and fast-spiking neurons. ***C***, LSPS-induced currents recorded from pyramidal (left) and fast-spiking neurons (right) shown in ***A***, ***B***. Evoked currents are arranged in the same sequence shown in ***A***. ***D***, A representative transsynaptic current evoked by laser photostimulation. Charge transfer from 5 to 70 ms (green area) was quantified to construct the excitation map. ***E***, Excitation maps obtained from pyramidal and fast-spiking neurons shown in ***A***–***C***. Direct responses (white pixels) occurring without latency following LSPS application were not included in the analysis.

A laser scanning photostimulation (LSPS) setup was built according to previously reported principles (Deleuze and Huguenard, 2006; Figs. 6, 7). LSPS was performed using an UV laser beam (349-nm wavelength, frequency-tripled Nd:YLF, 1-kHz pulse; Explorer One, Spectra-Physics) that was directed into a port of the confocal microscopic system (FV-1000, Olympus) and a back aperture of a 4× UPLSAPO objective (Olympus) equipped with a custom-made water-immersible cap. The position of the UV laser beam was adjusted with a galvanometer mirror controlled by FV-1000 software (FV-10, Olympus). The cue for the mirror movement and laser irradiation was TTL pulses generated by Digidata 1440A controlled by Clampex (Molecular Devices). Preincubation with recirculating high divalent cation (HDC) ACSF (4 mM Mg^2+^ and Ca^2+^) was performed for 6–10 min at the beginning of each experiment (Kumar et al., 2007). D-AP5 (25 μM; Tocris) and 4-methoxy-7-nitroindolinyl-caged-L-glutamate (MNI-caged-L-glutamate; Tocris; 200 μM) were added in 30-ml HDC ACSF.

To measure the laser beam power through a 4× objective, a UV-sensitive light power meter was used (power meter console, PM100D; probe, S130VC, Thorlabs). MNI-caged-L-glutamate was activated by a 5- to 6-mW UV pulse (2 kHz, 900 µs). To determine the optimized strength of the UV laser power, direct irradiations to the soma of the recorded pyramidal neurons in IC were performed, and the thresholds of the laser-evoked action potentials were evaluated (5–6 mW; Fig. 6). The spatial pattern of the UV laser stimulation consisted of 336 positions at ∼60-µm intervals, resulting in the coverage of the area of a vertically oriented grid 1750 µm along the mediolateral axis and horizontally oriented grid 720 µm along the pial-white matter axis. We used a random stimulation sequence pattern with a 4.5-s interstimulus interval. This pattern was carefully designed to ensure a distance of sequentially stimulating positions ≥120 µm. The responses evoked by the UV laser stimulation were analyzed within a time window of 5–70 ms and converted into a charge transfer. Responses with a short latency (<5 ms) and a long decay were identified as direct responses and were removed from the following analysis.

### Miniature IPSC (mIPSC) recording

We recorded the mIPSCs from Layer II/III pyramidal neurons in IC following the application of 40 μM DNQX, 50 μM D-AP5, and 1 μM TTX. The holding potential was set at +10 mV, which is near the reversal potential of glutamatergic inputs.

The other compounds were purchased from Wako Pure Chemical and Nakalai Tesque.

### Experimental design and statistical analyses

For the VSD imaging, 58 male rats were randomly assigned to the following five groups: 1w-sham (*N* = 15), 4w-sham (*N* = 10), 1w-IANX (*N* = 11), 2w-IANX (*N* = 12), and 4w-IANX (*N* = 10). In the comparison of the 1w-sham and 4w-sham groups, we did not find any differences in the responses to the dental pulp stimulations (Figs. 2, 3). Thus, we considered the 1w-sham and 4w-sham rats the sham control group (sham; *N* = 25), and their data were compared to those of the 1w-IANX, 2w-IANX, and 4W-IANX groups. Either Student’s *t* test or Mann–Whitney *U* test was used in accordance with the results of the normality test (Shapiro–Wilk test) and equal variance test (Brown–Forsythe test) to compare the peak amplitude, latency, time to peak, and area at peak. Similarly, paired *t* test was used to compare the peak amplitude of S1 and S2/IOR after the normality test. In the two group comparisons, a *p* < 0.05 was considered significant. For the comparison of the sham and 1w-INAX, 2w-INAX, and 4w-INAX groups, we used a Bonferroni correction, and *p* ≤ 0.017 was considered significant.

We used 11 male and 3 female VGAT-Venus transgenic rats without IANX (1w-sham) and 11 male VGAT-Venus transgenic rats with 1w-INAX for the Ca^2+^ imaging experiments. In the 1w-sham group, we obtained 178 responding and 468 nonresponding neurons from 11 male rats and 42 responding and 142 nonresponding excitatory neurons from three female rats. The ratio of responding to nonresponding neurons was comparable between the male and female rats (χ^2^(1) = 1.410, *p* = 0.235, χ^2^ test). We obtained 45 responding and 86 nonresponding inhibitory neurons from 11 male rats and 11 responding and 23 nonresponding inhibitory neurons from three female rats. The ratio of responding to nonresponding inhibitory neurons was comparable between the male and female rats (χ^2^(1) = 0.000256, *p* = 0.987, χ^2^ test). Therefore, we combined the data obtained from the male and female rats. A Kolmogorov–Smirnov test was used to compare the distribution patterns of the peak amplitude of the Ca^2+^ responses. A χ^2^ test was used to compare the rates of the responding neurons between the 1w-sham and 1w-IANX groups. A Mann–Whitney *U* test was used in accordance with the results of the normality test and equal variance test to compare the peak amplitude, time to peak, latency, rise time, and duration above 2 SD. We excluded nonresponding neurons from the analyses of the temporal kinetics of the Ca^2+^ responses between the 1w-sham and 1w-IANX groups. A *p* < 0.05 was considered significant.

**Figure 8.**
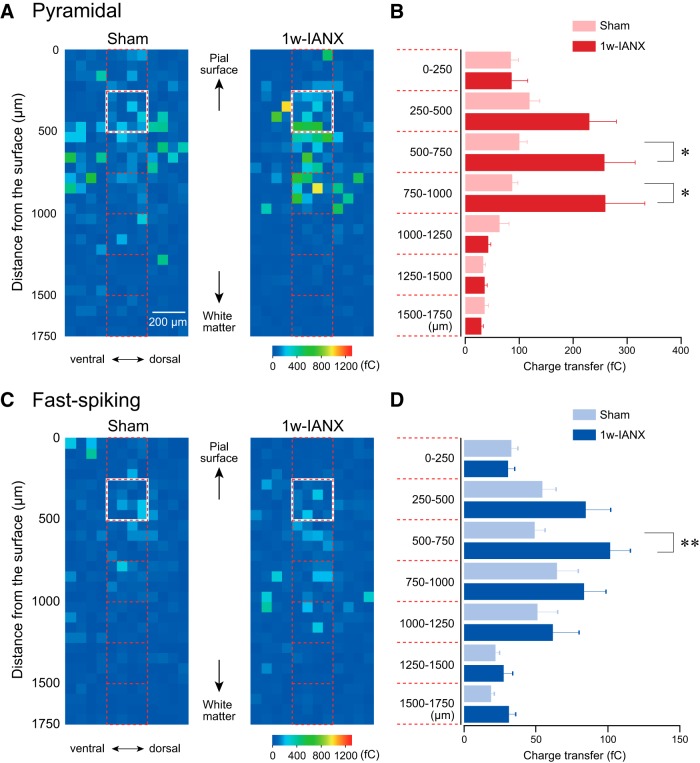
Averaged LSPS-induced excitation maps obtained from pyramidal and fast-spiking neurons in Layer II/III IC of sham and 1w-IANX rats. ***A***, Excitation maps obtained from pyramidal neurons in sham (*n* = 14; left) and 1w-IANX (*n* = 18; right) rats. The maps were aligned to the reference of the line of the pial surface and vertical axis perpendicular to the pial surface. Recorded pyramidal neurons are located within the white quadrangles. Note that pyramidal neurons receive dense excitatory inputs from deeper layers that correspond to Layer IV and superficial Layer V. ***B***, Averaged charge transfer obtained from area enclosed by red quadrangles shown in ***A*** was compared. The charge transfer from pyramidal neurons in the 1w-IANX group was larger than that in the sham group at depths from 500 to 1000 μm from the pial surface. ***C***, Excitation maps obtained from fast-spiking neurons in the sham (*n* = 19; left) and 1w-IANX (*n* = 10; right) groups. Recorded fast-spiking neurons are located within the white quadrangles. Note that fast-spiking neurons receive dense excitatory inputs from deeper layers that correspond to Layer IV and superficial Layer V. ***D***, Averaged charge transfer of fast-spiking neurons in the 1w-IANX group is larger than that in the sham group at depths from 500 to 750 μm from the pial surface; *p < 0.05, **p < 0.01, Student’s *t* test.

In the *in vitro* whole-cell patch-clamp recording studies, 36 VGAT-Venus transgenic rats were used. Thirty rats were assigned to the caged glutamate experiments, and six rats were used for the mIPSC recording. The differences in the excitatory input between the 1w-sham and 1w-IANX groups were evaluated by the averaged charge transfer (16 panels, 250 × 250 µm) centered on a middle line along the vertical axis (Fig. 8). Student’s *t* test was used to compare the charge transfer of the evoked EPSCs between the 1w-sham and 1w-IANX groups. Student’s *t* test or Mann–Whitney *U* test were used to compare the interevent interval and amplitude of the mIPSCs between the 1w-sham and 1w-IANX groups; *p* < 0.05 was considered significant.

The data are expressed as the mean ± SEM. In Results, *n* refers to the number of recorded neurons or slices, and *N* refers to the number of animals. The statistical analysis was performed by software programs (SigmaStat version 4.0, Systat Software; Origin version 8, OriginLab). Minor adjustments to the image brightness and contrast were performed in Adobe Photoshop (version CC 2015; Adobe Systems). The final schematic figures were generated using Adobe Illustrator (version CC 2015; Adobe Systems).

## Results

In the present study, first, we performed *in vivo* VSD imaging of S1 and S2/IOR, which process nociceptive information from the dental pulps, to examine the effects of IANX on the spatiotemporal properties of cortical excitation at a macroscopic level. Second, we investigated the mechanism of the changes in the cortical responses at the cellular level by *in vivo* Ca^2+^ imaging using two-photon microscopy. Finally, *in vitro* whole-cell patch-clamp recording was performed to evaluate whether the changes occur in cortical neural circuits and to examine the underlying mechanisms of the changes in the cortical circuits *in vivo*.

### Pulpal stimulation-induced optical responses in the sham groups

We performed VSD imaging in the 1w-sham and 4w-sham groups, which received an operation to expose the IAN without IANX. Similar to our previous studies in naïve rats (Nakamura et al., 2015, 2016), electrical stimulation of the mandibular or maxillary molar pulps independently induced excitation in S1 and S2/IOR, and the excitation propagated in a concentric manner from the initially excited regions (Figs. 2*A*, 3*A*). A large part of the areas activated by the maxillary and mandibular molar pulp stimulation in the S2/IOR overlapped (Figs. 2*D*, 3*D*).

The peak amplitude of the excitation at the center of the initial excitation in S2/IOR (mandibular molar pulp, 0.229 ± 0.027, *N* = 25; maxillary molar pulp, 0.186 ± 0.018, *N* = 25) was larger than that in S1 (mandibular molar pulp, 0.183 ± 0.036, *N* = 25, *t*_(24)_ = –3.190, *p* = 0.004, paired *t* test; maxillary molar pulp, 0.107 ± 0.015, *N* = 25, *t*_(24)_ = –6.938, *p* < 0.001, paired *t* test). The amplitude of the excitation in S2/IOR was comparable between the 1w-sham and 4w-sham groups [mandibular molar pulp, 0.240 ± 0.039, *N* = 15 in the 1w-sham group and 0.214 ± 0.033, *N* = 10 in the 4w-sham group, *U* = 73.0, *p* = 0.934, Mann–Whitney *U* test (Fig. 2*B*,*C*); maxillary molar pulp, 0.194 ± 0.023, *N* = 15 in the 1w-sham group and 0.174 ± 0.029, *N* = 10 in the 4w-sham group, *U* = 61.0, *p* = 0.454, Mann–Whitney *U* test (Fig. 3*B*,*C*)]. Other temporal kinetic characteristics of the excitation in S2/IOR, including the latency and time to peak, were also comparable between the 1w-sham and 4w-sham groups (Figs. 2*C*, 3*C*). Furthermore, the spatial patterns of the excitation in S1 and S2/IOR in the 4w-sham group were quite similar to those in the 1w-sham group (Figs. 2*A*,*E*, 3*A*,*E*). These results suggest that the development of excitatory propagation profiles in S1 and S2/IOR reaches a plateau by six to seven weeks of age. Thus, we considered the 1w-sham and 4w-sham groups a group of sham control rats (sham) for the comparisons of the spatiotemporal kinetics between the sham groups and IANX models.

### Recovery of cortical responses following mandibular molar stimulation from IANX

To examine the time course of excitation recovery from IANX, optical responses were recorded from 1w-IANX, 2w-IANX, and 4w-IANX rats (Fig. 1*E*).

The cortical excitation in response to the mandibular molar pulp stimuli was almost abolished in the 1w-IANX rats (Fig. 2) as follows: in 5/11 rats, no significant optical signal response was observed, and the other six rats showed only faint excitatory responses in S2/IOR. This faint response exhibited a smaller amplitude (0.229 ± 0.027 in the sham group and 0.085 ± 0.017 in the 1w-IANX group, *N* = 11, *U* = 29.0, *p* < 0.001, Mann–Whitney *U* test), a longer latency, and a smaller excited area (Fig. 2*C–F*). These results suggest that the IAN does not recover from transection within a week.

In contrast, 83.3% of 2w-IANX rats (10/12 rats) showed weak, but significant excitation (Fig. 2), although a significant optical signal was not observed in 18.2% of the 2w-IANX rats (2/12 rats). In summary, the peak amplitude in the 2w-IANX group recovered to 58% of that in the sham group (0.229 ± 0.027 in the sham group and 0.134 ± 0.013 in the 2w-IANX group, *N* = 12, *U* = 78.0, *p* = 0.020 vs sham group, Mann–Whitney *U* test; Fig. 2*C*). The latency and time to peak of excitation were comparable to those of the sham group (Fig. 2*C*). The excitation area almost recovered to the control level (8.49 ± 1.48 mm^2^ in the sham group and 8.00 ± 2.02 mm^2^ in the 2w-IANX group, *N* = 12, *U* = 142.0, *p* = 0.808 vs the sham group, Mann–Whitney *U* test; Fig. 2*D–F*). These results suggest that the 2w-IANX rats partially recovered as follows: a part of the transected IAN recovered and could conduct peripheral information from the mandibular molar pulp.

In the 4w-IANX group, the excitatory responses to the mandibular molar stimulation almost recovered to the control level (Fig. 2). There were no significant differences in the peak amplitude, latency, time to peak, and area at peak between the 4w-IANX group (*N* = 10) and sham group (*N* = 25, peak amplitude, *U* = 77.0, *p* = 0.083, Mann–Whitney *U* test; latency, *U* = 85.5, *p* = 0.136, Mann–Whitney *U* test; time to peak, *U* = 113.5, *p* = 0.687, Mann–Whitney *U* test; area at peak, *U* = 115.0, *p* = 0.729, Mann–Whitney *U* test; Fig. 2*C*,*E*).

These findings suggest that the transected IAN recovers within four weeks. This time course is consistent with a previously published behavioral study (Hitomi et al., 2012).

### Facilitative excitation responses to maxillary molar stimulation in IANX model rats

Nociceptive information from the mandibular and maxillary molar pulps converges in S1 and S2/IOR, and these excitatory regions partially overlap (Nakamura et al., 2015, 2016). Therefore, the IANX-induced imbalance of the nociceptive inputs from these molar pulps could induce plastic changes in cortical excitation as follows: the excitatory region responding to the maxillary molar stimulation may expand because the maxillary nerve is intact in the IANX model rats. To examine this possibility, the optical responses to maxillary molar stimulation were examined in 1w-IANX, 2w-IANX, and 4w-IANX rats (Fig. 3).

In contrast to the abolished response to the mandibular molar stimulation observed in the 1w-IANX rats, the cortical excitation in response to the maxillary molar stimulation was potently expanded compared with that in the sham group (Fig. 3*A*,*B*). The peak amplitude increased from 0.196 ± 0.018 to 0.367 ± 0.069 (sham group, *N* = 25; 1w-IANX group, *N* = 11, *U* = 58.0, *p* = 0.007, Mann–Whitney *U* test; Fig. 3*C*), which was accompanied by a shortened latency (17.5 ± 1.5 ms in the sham group to 12.5 ± 0.6 ms, *U* = 137.0, *p* = 0.016, Mann–Whitney *U* test) and time to peak (49.2 ± 5.1 ms in the sham group to 26.0 ± 2.8 ms, *U* = 55.5, *p* = 0.005, Mann–Whitney *U* test). The changes in the peak amplitude, latency, and time to peak recovered to the control level in the 2w-IANX and 4w-IANX rats (Fig. 3*C*).

In addition to the changes in the temporal profiles of the optical signal, the area of cortical excitation induced by the maxillary molar stimulation was expanded one week after IANX (5.1 ± 0.6 to 14.3 ± 2.7 mm^2^, *U* = 51.0, *p* = 0.003, Mann–Whitney *U* test; Fig. 3*D*,*E*). The expanded area of excitatory propagation in S2/IOR in the 1w-IANX group was observed dorsally adjacent to that in the sham group (Fig. 3*F*). This facilitation reached the maximum in the 1w-IANX group, and the 2w-IANX rats still showed an enhanced excitatory propagation compared to that in the sham group (5.1 ± 0.6 mm^2^ in the sham group to 10.0 ± 1.6 mm^2^ in the 2w-IANX group, *U* = 76.0, *p* = 0.017, Mann–Whitney *U* test). The rate of enhancement in the 2w-IANX group was smaller than that in the 1w-IANX group as shown in Figure 3*D*,*E*. In parallel to the recovery of the cortical response to the mandibular molar stimulation (Fig. 2), the facilitative excitatory propagation in response to the maxillary molar stimulation recovered to the control level four weeks after IANX (Fig. 3).

### Neural responses to molar stimulation revealed by Ca^2+^imaging

The increase in the optical signal responses to the maxillary molar pulpal stimulation implies that a plastic change occurred in the cortical circuits. However, the mechanisms of the IANX-induced facilitation of cortical responses at the cellular level cannot be clarified by *in vivo* optical imaging using a VSD. Ca^2+^ imaging using a two-photon microscope enabled us to detect the neural activities in each neuron and identify the types of neurons, i.e., excitatory glutamatergic or inhibitory GABAergic neurons. A critical question for understanding the facilitative mechanisms induced by IANX is determining which types of neurons are more sensitive to IANX. Thus, we performed *in vivo* Ca^2+^ imaging in the focused region of S2/IOR (Fig. 4*B*,*C*) in sham and 1w-IANX rats to examine the types of cells facilitated by IANX.

First, we systematically imaged the Venus fluorescence from the surface of the IC to a depth of 400 μm to discriminate the inhibitory neurons from the excitatory neurons (Fig. 4*D*). Then, a mixture of a Ca^2+^ indicator, i.e., OGB_488_, and an astrocyte marker, i.e., SR-101, was injected to the imaged area in S2/IOR to measure the Ca^2+^ responses and distinguish the source of the Ca^2+^ signals, i.e., neurons or astrocytes (Fig. 4*E*). Serial imaging of OGB_488_ was performed before, during, and after the electrical stimulation of the maxillary molar pulp (Fig. 4*A*). A *post hoc* analysis was performed using superimposed images of Venus and OGB_488_/SR-101, and the recorded cells were classified as Venus-negative and SR-101-negative cells, Venus-positive and SR-101-negative cells, and Venus-negative and SR-101-positive cells, which are considered glutamatergic excitatory neurons, GABAergic inhibitory neurons, and astrocytes, respectively (Fig. 4*F*). In this study, Ca^2+^ responses in astrocytes were excluded from the following analysis.

The electrical stimulation of the maxillary molar pulp increased the Ca^2+^ signals in both the excitatory and inhibitory neurons (Fig. 4*Ga*,*b*). Even in the same recorded area, some neurons did not show significant Ca^2+^ responses in the sham group (Fig. 4*Gc*). Similarly, the 1w-IANX rats exhibited Ca^2+^ responses to the maxillary molar pulp stimulation in both excitatory and inhibitory neurons (Fig. 4*Ha*,*b*), with some exceptions showing an undetectable increase in Ca^2+^ signals (Fig. 4*Hc*).

The *post hoc* analysis of the Ca^2+^ responses revealed that the kinetics differed between the sham and 1w-IANX groups. In the excitatory neurons, the mean of the peak amplitude increased from 2.21 ± 0.04 to 2.50 ± 0.04 (sham group, *n* = 830, *N* = 14, 1w-IANX group, *n* = 818, *N* = 11, *U* = 282485.0, *p* < 0.001, Mann–Whitney *U* test). In the inhibitory neurons, the mean of the peak amplitude increased from 2.28 ± 0.09 to 2.96 ± 0.10 (sham group, *n* = 165, *N* = 14, 1w-IANX group, *n* = 178, *N* = 11, *U* = 10173.0, *p* < 0.001, Mann–Whitney *U* test). The cumulative curves of the peak Ca^2+^ response amplitudes in the excitatory and inhibitory neurons of the 1w-IANX group shifted rightward compared to those in the sham group (*p* < 0.001, Kolmogorov–Smirnov test; Fig. 5*A*). The number of responding excitatory neurons increased from 220 of 830 to 297 of 818 (26.5–36.3%, χ^2^(1) = 17.933, *p* < 0.001, χ^2^ test; Fig. 5*B*). Similarly, the number of responding inhibitory neurons increased from 56 of 165 to 105 of 178 (33.9–59.0%, χ^2^(1) = 20.578, *p* < 0.001, χ^2^ test). These findings of increased peak amplitude and rate of responding excitatory and inhibitory neurons are consistent with the enhancement of cortical excitation revealed by the VSD imaging described above (Fig. 3).

Interestingly, the excitatory neurons showed a longer latency and prolonged duration compared to those in the sham group (Fig. 5*D*,*F*) as follows: latency increased from 167.6 ± 12.0 ms (*n* = 220, *N* = 14) to 190.5 ± 10.0 ms (*n* = 297, *N* = 11; *U* = 28735.0, *p* = 0.019; Mann–Whitney *U* test), and duration twice above the SD increased from 348.0 ± 18.0 ms to 402.8 ± 19.3 ms (*U* = 29234.0, *p* = 0.041; Mann–Whitney *U* test). In addition, in both the excitatory and inhibitory neurons in the 1w-IANX rats, the time to peak of the Ca^2+^ increase was significantly larger than that in the Sham rats (excitatory, *U* = 26620.5, *p* < 0.001, Mann–Whitney *U* test; inhibitory, *U* = 2262.0, *p* = 0.016, Mann–Whitney *U* test; Fig. 5*C*). Similarly, the 20–80% rise time in the 1w-IANX was significantly larger than that in the sham rats (excitatory, *U* = 26639.0, *p* < 0.001, Mann–Whitney *U* test; inhibitory, *U* = 2183.5, *p* = 0.007, Mann–Whitney *U* test; Fig. 5*E*). These results suggest that the enhancement of neural excitation is likely due to sustained spike firing in response to the electrical stimulation of the maxillary molar pulp.

### Action potential induction by LSPS

The facilitation of neural activities in Venus-negative non-GABAergic neurons and Venus-positive GABAergic neurons is controversial because the facilitation of inhibitory GABAergic neuronal activities may further suppress excitation in surrounding neurons. To elucidate this discrepancy, it is necessary to clarify how excitatory projections to non-GABAergic and GABAergic neurons in Layer II/III, representing a principal source of optical signals, are changed in 1w-IANX rats. Furthermore, we are interested in what changes occur in inhibitory synaptic transmission onto non-GABAergic pyramidal neurons. Thus, we examined the changes in excitatory inputs to Layer II/III pyramidal and fast-spiking neurons by LSPS-induced uncaging of caged-glutamate to evaluate the excitatory connections in IC slice preparations (see Materials and Methods).

We evaluated the strength of the laser intensity that induced an action potential in non-GABAergic pyramidal, presumably glutamatergic excitatory, neurons in IC slice preparations by uncaging the caged-glutamate (200 µM) applied to HDC ACSF. A representative fluorescent image of a pyramidal neuron visualized with Alexa Flour 594 and laser-shooting points at 10-µm intervals (blue circles) are shown in Figure 6*A*. First, the point at the center of the soma was photostimulated (5-ns duration, four pulses at 5 kHz) at 1.0, 2.3, and 4.6 mW. Although 1.0- and 2.3-mW LSPS did not evoke an action potential (Fig. 6*Ba*,*b*), 4.6-mW LSPS evoked a single action potential with a 5-ms latency (Fig. 6*Bc*). Figure 6*C* shows a summary of the results of the detection of the threshold of the photostimulation intensity that evokes an action potential (*n* = 11 neurons in *n* = 11 slice preparations, *N* = 9). Although 5.2-mW LSPS invariably evoked action potentials, this intensity often evoked multiple action potentials in some neurons, which may have evoked postsynaptic potentials via multiple synapses. To avoid this possibility, we set the stimulation intensity at 5.1 mW, which evoked an action potential in 91% neurons. The latency of the action potentials was measured following the application of photostimulation 5.1 mW (Fig. 6*D*). More than 95% of the pyramidal neurons showed an action potential within 70 ms (97.7%), and therefore, the temporal time window for the analysis of the LSPS-induced synaptic responses was set at 5–70 ms. Figure 6*E* shows the locations of the action potentials induced by LSPS (5.1 mW) in the same neuron shown in Figure 6*A*,*B*. A single action potential was induced in the cases in which LSPS was applied to the soma and apical dendrite. The averaged map indicates the probability of action potential induction, demonstrating the low probability of action potential induction in response to LSPS at distal sites (Fig. 6*F*,*G*). The bath application of TTX (1 µM) with caged-glutamate (200 µM) blocked action potential induction but not LSPS-evoked remaining depolarization, which is a direct response that was abolished by 40 µM DNQX (Fig. 6*H*). This suggests that depolarization by direct glutamate application to the recorded neuron underlies the action potentials evoked by LSPS.

### Changes in excitatory connections to Layer II/III pyramidal and fast-spiking neurons in IC

Figure 7 shows an example of a simultaneous recording from a pyramidal neuron and a fast-spiking neuron in IC Layer II/III. The pyramidal neuron was Venus-negative and showed spike adaptation in response to a long depolarizing current pulse injection, whereas the fast-spiking neuron was Venus-positive and showed repetitive spike firing at a high frequency without spike adaptation (Fig. 7*B*). LSPS was applied to the grid (336 points) spacing at a 62.5-μm interval that covered almost the whole IC layers (750 μm in width and 1750 μm in length). LSPS induced inward currents in some grids (Fig. 7*C*). The evoked responses were classified into the following two categories: direct responses evoked without latency that were often accompanied by action currents, and synaptic responses induced with a 5- to 70-ms latency (see Materials and Methods). We estimated the latter responses to evaluate the strength of the excitatory projection from each grid to the recorded neurons by measuring the charge transfer of the responses (Fig. 7*D*).

The color-coded charge transfer at each stimulation site was tiled as an activation map (Fig. 7*E*). The direct responses were principally located around the soma of the recorded neurons, while the synaptic responses were widely distributed throughout the slice preparation. The maps obtained from each slice preparation were superimposed to the reference of the somata of the recorded neurons, edge of the cortical surface, and the vertical axis perpendicular to the cortical surface. The averaged charge transfer in each grid was averaged and color-coded (Fig. 8). In the sham group, the pyramidal neurons in Layer II/III received excitatory synaptic inputs from deeper layers corresponding to Layer IV and upper Layer V (*n* = 14, *N* = 6; Fig. 8*A*, left). This layer specificity of excitatory inputs to Layer II/III pyramidal neurons was maintained in the 1w-IANX group (*n* = 18, *N* = 6; Fig. 8*A*, right); however, the averaged charge transfer of the synaptic responses from Layers VI and V (500–1000 μm in depth from the surface) was significantly larger than that in the sham group as follows: in the depth of 500–750 µm, 99.9 ± 14.3 fC in the sham group and 257.6 ± 55.3 fC in the 1w-IANX group (*t*_(30)_ = –2.672, *p* = 0.012, Student’s *t* test), and in the depth of 750–1000 µm, 86.8 ± 10.2 fC in the sham group and 259.2 ± 71.1 fC in the 1w-IANX group (*t*_(30)_ = –2.324, *p* = 0.027, Student’s *t* test; Fig. 8*B*).

Similar to the pyramidal neurons, the fast-spiking neurons in the sham group received dense excitatory inputs from the deeper layers (Fig. 8*C*, left). Similar to the increased excitatory synaptic inputs to the pyramidal neurons in the 1w-IANX group, the fast-spiking neurons received larger excitatory synaptic inputs principally from the deeper layers in the 1w-IANX group (*n* = 10, *N* = 7; Fig. 8*C*, right) compared to those in the sham group [*n* = 19, *N* = 14 (Fig. 8*C*, left); in the depth of 500–750 µm, 49.3 ± 7.0 fC in the sham group and 102.0 ± 13.7 fC in the 1w-IANX group (*t*_(30)_ = 3.311, *p* = 0.002, Student’s *t* test (Fig. 8*D*)].

These results suggest that Layer II/III pyramidal and fast-spiking neurons in the 1w-IANX group receive larger excitatory inputs from deeper layers than those in the sham group. These facilitative excitatory inputs likely underlie the enhancement of excitatory propagation and larger amplitude of Ca^2+^ signals with more responding neurons in the 1w-IANX group as revealed by the VSD imaging and Ca^2+^ imaging, respectively.

### Reduction in inhibitory inputs to pyramidal neurons

To evaluate the efficacy of the inhibitory synaptic transmission to the pyramidal neurons, we recorded mIPSCs following the application of 40 μM DNQX, 50 μM D-AP5, and 1 μM TTX, which are AMPA and NMDA receptor antagonists, and a voltage-gated sodium channel blocker, respectively. We used a Cs-based internal solution, and the holding potential was set at +10 mV to isolate the GABA_A_ receptor-mediated IPSCs.

**Figure 9.**
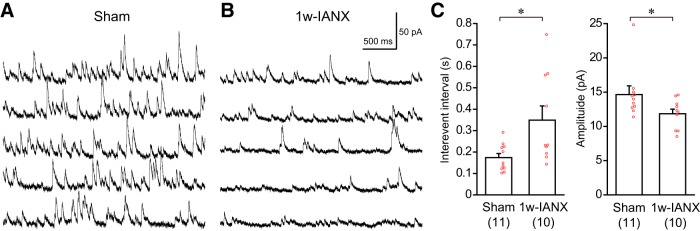
Representative examples of mIPSCs recorded from Layer II/III pyramidal neurons in the sham and 1w-IANX groups. ***A***, Continuous mIPSC recording in sham rats following the application of 40 μM DNQX, 50 μM D-AP5, and 1 μM TTX. Holding potential was set at +10 mV. ***B***, Continuous mIPSC recording in 1w-IANX rats. ***C***, Comparison of interevent interval and amplitude of mIPSCs between the sham (*n* = 11) and 1w-IANX (*n* = 10) groups. Note the significant increase in the interevent interval and decrease in amplitude in the 1w-IANX group; *p < 0.05, Student’s *t* test for interevent interval and Mann–Whitney *U* test for amplitude.

Typical examples of mIPSCs from one 1w-sham and one 1w-IANX rats are shown in Figure 9*A*,*B*, respectively. The application of 100 μM picrotoxin diminished the mIPSCs (data not shown), indicating that the mIPSCs were mediated by GABA_A_ receptors. The interevent interval of the mIPSCs in the 1w-IANX group was larger than that in the sham group (sham group, 0.17 ± 0.02 s, *n* = 11, *N* = 4; 1w-IANX group, 0.35 ± 0.07 s, *n* = 10, *N* = 2; *t*_(19)_ = 2.644, *p* = 0.016, Student’s *t* test; Fig. 9*C*). In addition, the mIPSC amplitude in the 1w-IANX group was smaller than that in the sham group (sham group, 14.6 ± 1.3 pA, *n* = 11, *N* = 4; 1w-IANX, group 11.9 ± 0.7, *n* = 10, *N* = 2; *U* = 24.0, *p* = 0.032, Mann–Whitney *U* test; Fig. 9*C*). These results suggest that IANX reduces inhibitory synaptic inputs onto pyramidal neurons in IC Layer II/III.

## Discussion

We focused on the plastic changes in excitation in S2/IOR in IANX model rats in which the mandibular but not maxillary nerve branch was transected. Optical imaging using VSD revealed contradictory excitatory responses one week after IANX between mandibular and maxillary molar pulp stimulation as follows: the loss of excitation following mandibular stimulation versus facilitated excitation following maxillary stimulation. At this stage, the neural activities in Layer II/III excitatory and inhibitory neurons in S2/IOR in the IANX model rats were enhanced due to facilitated glutamatergic inputs. On the other hand, the inhibitory inputs onto the pyramidal neurons were reduced in the IANX model rats.

### Which brain region is responsible for the IANX-induced enhancement of the maxillary molar region?

Primary afferents are considered electrically isolated; however, their somata in the TG communicate each other via nitric oxide signaling (Sugiyama et al., 2013) and satellite glial cells expressing connexin 43, which is the basis for mutual electrical coupling (Kaji et al., 2016). These couplings between TG neurons contribute to the spreading of intracellular signaling from injured afferents, i.e., the transected IAN, to adjacent nerves. Consequently, several ionic channels, including TTX-sensitive and resistant Na^+^ channels, are overexpressed, and transient and sustained K^+^ currents are decreased in the maxillary nerves (Nakagawa et al., 2010), decreasing the threshold of noxious stimuli and increasing responses to thermal stimulation (Iwata et al., 2001; Saito et al., 2008).

In addition to TG, IANX increases the background activity and hyperexcitation of secondary neurons in Sp5C in response to mechanical and thermal stimuli, lasting for at least two weeks after IANX (Iwata et al., 2001; Saito et al., 2008). Okada-Ogawa et al. (2015) demonstrated a reduction in the number of GABAergic neurons and a reduced expression of K^+^-Cl^–^ co-transporter 2 in Sp5C of IANX model rats. Glial mechanisms are also involved in extraterritorial neuropathic pain in IANX models (Okada-Ogawa et al., 2009).

At the sensory thalamic level, IANX changes the profiles of neural firing. Neurons in VPM responding to the maxillary nerve division show increases in spontaneous firing and mechanical stimulation-induced responses (Tseng et al., 2012). In addition, these VPM neurons exhibit an expanded receptive field, a modality shift, and long-lasting after-discharges (Tseng et al., 2012).

In contrast to these subcortical regions, limited information is available regarding the cerebral cortex after IANX. The present study is the first to demonstrate that neuroplastic changes certainly occur in the cerebral cortex. Our slice experiment, which excluded the effects of subsequent activity changes in subcortical regions, demonstrated that the local IC circuit certainly changes in IANX models. Therefore, changes in cortical circuits are a part of the mechanisms responsible for cortical hyperactivities.

### Facilitative mechanisms of cortical excitation induced by IANX

Plastic changes in the central nervous system indicate that recovery of the injured peripheral nerves is not sufficient for the suppression of abnormal pain. To develop a new clinical approach for the suppression of neuropathic pain, it is necessary to understand the mechanisms of abnormal excitation in the cerebral cortex.

The number of inhibitory neurons responding to the maxillary molar pulp stimulation was approximately two times greater in the 1w-IANX group than that in the sham group. In addition, the peak amplitude of the Ca^2+^ responses was also increased in the IANX rats. However, the 1w-IANX excitatory neurons also exhibited increases in the rise time, time to peak, and duration of Ca^2+^ responses, suggesting that the facilitation of inhibitory neurons cannot suppress the increase in neural activities in 1w-IANX excitatory neurons. The facilitative activities of excitatory and inhibitory neurons in the 1w-IANX group revealed by *in vivo* Ca^2+^ imaging are supported by the *in vitro* finding that both non-GABAergic pyramidal neurons and GABAergic neurons in IC slice preparations obtained from the 1w-IANX group received significantly larger excitatory inputs than those of the sham group. These contradictory results may be explained by the increase in the interevent interval and the decrease in the mIPSC amplitude recorded from the pyramidal neurons.

The longer latency of the Ca^2+^ responses in the 1w-IANX excitatory neurons compared to that in the Sham neurons seems to contradict other results supporting the facilitation of neural excitation in the 1w-IANX group. Newly recruited neurons by IANX may receive smaller excitatory inputs than originally responding neurons; therefore, the latency could be longer than that in the sham group.

A reduced potency of inhibitory neurons could be a common phenotype in pain models. A previous study (Eto et al., 2012) reported that the activities of excitatory and inhibitory neurons in S1 are increased in an inflammatory chronic pain model induced by an injection of complete Freund’s adjuvant (CFA) into the hind paw of mice. In the CFA model, a reduction in the expression and function of K^+^-Cl^–^ co-transporter 2 in excitatory neurons was observed without a change in the release probability of GABA and the number of postsynaptic GABA_A_ receptors (Eto et al., 2012). However, our whole-cell patch-clamp recording suggests a reduction in the release probability with changing postsynaptic GABA_A_ receptor properties in the 1w-IANX group. Thus, the local circuit mechanisms may differ among pain models and cortical areas.

### Temporal profiles of changes in neural excitation induced by IANX

Hyperexcitability is observed in injured primary afferents 2 h after IANX and lasts for 30 d (Bongenhielm and Robinson, 1996, 1998; Chudler et al., 1997; Tseng et al., 2012). Although an early onset of changes in thalamic activities has been reported in a sciatic nerve injury model (Brüggemann et al., 2001; Komagata et al., 2011), Tseng et al. (2012) reported that thalamic facilitation is observed 7–30 d after IANX but not within 3 d and that hyperexcitation in TG and VPM neurons terminates 40–60 d after IANX. The temporal profiles of the cortical hyperexcitability in this study are almost consistent with the temporal profile of the VPM. The delay of the onset of hyperexcitability suggests that the plastic changes sequentially occur from the periphery to higher brain regions. A possible mechanism of the delayed onset is that the plastic changes in the higher order brain regions require the facilitation of bottom-up activities.

### Functional implications

Nerve injury in the oral region often causes ectopic pain in the surrounding region (Bender, 2000). The somatotopic organization of S1 and S2 may explain the mechanisms of this ectopic pain. Our previous studies have demonstrated that S2/IOR principally mediates nociception in the orofacial region (Horinuki et al., 2015, 2016; Nakamura et al., 2015, 2016; Kaneko et al., 2017). Notably, IANX expanded the excitation in S2/IOR toward the dorsal regions because the region dorsally adjacent to S2/IOR corresponds to facial S1 and S2, including the whisker pad (Remple et al., 2003). Therefore, our finding of cortical remodeling in S2/IOR is likely a component of the mechanism of ectopic pain induced by IANX.

Medicines affecting inhibitory neural systems, such as benzodiazepine, are used to clinically relieve abnormal pain (Reddy and Patt, 1994; Baillie and Power, 2006; Kukkar et al., 2013; Howard et al., 2014). This effect is considered indirect; the anxiolytic and muscle-relaxant effects of benzodiazepine play a role in relieving abnormal pain (Reddy and Patt, 1994; Howard et al., 2014). We consider these medicines directly effective in suppressing abnormal cortical excitation in orofacial neuropathic pain because one possible explanation for the facilitated neural excitation in S2/IOR was a reduction in GABAergic inputs to pyramidal neurons.

## Synthesis

Reviewing Editor: Karen Davis, Krembil Research Institute and University of Toronto Decisions are customarily a result of the Reviewing Editor and the peer reviewers coming together and discussing their recommendations until a consensus is reached. When revisions are invited, a fact-based synthesis statement explaining their decision and outlining what is needed to prepare a revision will be listed below. The following reviewer(s) agreed to reveal their identity: Qian-Quan Sun. Note: If this manuscript was transferred from JNeurosci and a decision was made to accept the manuscript without peer review, a brief statement to this effect will instead be what is listed below.

The authors has done a good job to address the previous reviewer concerns.

However, there are some minor grammatical and spelling errors, which need the authors to go through and make changes.
